# Comparison of baseline quality of life measures between renal cell carcinoma patients undergoing partial versus radical nephrectomy

**DOI:** 10.1186/1471-2490-13-52

**Published:** 2013-10-22

**Authors:** Michelle L Arnold, David D Thiel, Nancy Diehl, Kevin J Wu, Steve Ames, Alexander S Parker

**Affiliations:** 1 Department of Health Sciences Research, 4500 San Pablo Road, Jacksonville, FL 32224, USA; 2 Department of Urology, 4500 San Pablo Road, Jacksonville, FL 32224, USA; 3 Department of Anatomic Pathology, 4500 San Pablo Road, Jacksonville, FL 32224, USA; 4 Department of Medicine at Mayo Clinic, 4500 San Pablo Road, Jacksonville, FL 32224, USA

**Keywords:** Kidney cancer, Quality of life, Radical nephrectomy, Nephron-sparing surgery

## Abstract

**Background:**

To compare demographics, pathologic features, performance scores, comorbidities, symptoms and responses to quality of life (QoL) surveys between nephron-sparing surgery (NSS) and radical nephrectomy (RN) patients prior to surgical intervention. Previous investigators have compared QoL outcomes for patients undergoing RN and NSS; however, there are limited data comparing QoL-related characteristics at baseline between these groups.

**Methods:**

We identified 144 patients with localized RCC who underwent either NSS (n = 71) or RN (n = 73) between May ‘07-November ‘12. We abstracted baseline data on demographic and clinic-pathologic variables as well as responses to the SF-36 and FACT-G surveys from our prospective registry. We amended the FACT-G with 8 additional questions designed to address RCC-specific QoL. For comparisons between the two groups, we employed Wilcoxon rank-sum and Fisher's Exact tests where appropriate.

**Results:**

We observed RN patients to have more aggressive pathology. We noted no difference in performance scores between the two groups; however, RN patients were more likely to have higher Charlson scores (p = 0.022) and various symptoms at presentation (all p <0.001). For the QoL surveys, we did not observe differences on the FACT-G; however, we noted evidence of differential scores between the two groups on specific domains of the SF-36 (e.g. Mental Health; p 0.022) and the RCC-specific QoL questions added to the FACT-G.

**Conclusions:**

We report baseline differences between RN and NSS patients on clinico-pathologic as well as QoL-related metrics. As issues of survivorship become increasingly important, our results underscore the need to consider baseline status in evaluations of QoL-related outcomes for patients undergoing surgery for RCC.

## Background

The standard treatment for localized, unilateral renal cell carcinoma (RCC) is surgical excision. Related to this, while radical nephrectomy (RN) remains the best option for many RCC patients, nephron-sparing surgery (NSS) has evolved over the past two decades into the standard treatment for patients presenting with pT1a RCC and its use is increasing for pT1b and pT2 RCC patients as well [1,2]. The emergence of NSS as a viable means of maintaining cancer control for patients with localized RCC has generated interest in the broader question of whether NSS and RN have similar effects on the post-surgical quality of life (QoL). In an age where RCC incidence is rising [3] and issues of cancer survivorship have become germane, a better understanding of the effect of NSS compared to RN on standard QoL metrics has the potential to further inform the shared decision-making process for surgeons and patients faced with surgical management options for clinically localized RCC.

To date, a handful of investigators have addressed the question of QoL outcomes in RCC patients [4-10]; however only three have directly addressed the direct comparison of QoL measures between patients undergoing NSS and RN [5,6,8]. Interestingly, while one study reported no difference in QoL-related outcomes between the two surgical groups [5], two of the studies presented evidence suggesting improved QoL outcomes for the NSS versus RN group [6,8]. A key limitation in each of these investigations is the absence of information on baseline measures of QoL prior to surgery. More recently Parker et al. [10] advanced the field with their report from the largest and most definitive observational study to date on the issue of QoL outcomes across four common surgical interventions. In contrast to previous studies, the authors described their access to baseline QoL measures prior to surgical intervention; however, they did not present these baseline data for review and did not adjust for baseline status in their analyses, citing only a lack of statistically significant difference between the groups at baseline. Moreover, the authors do not discuss baseline comparisons on QoL-related metrics such as presence of comorbidities, performance score or symptomatic presentation. As such, a simple yet important question that remains unclear is whether RCC patients undergoing NSS and RN have similar baseline levels of QoL and other related metrics prior to surgical intervention.

Motivated by this continuing gap in our knowledge, we explore for the first time whether patients who undergo NSS and those that undergo RN have similar QoL status at baseline. Specifically, we hypothesize that there are key differences between RN and NSS patients at baseline and as such, these should be taken in to account when comparing follow-up outcomes between these two groups. To test this hypothesis, we compare baseline demographics, pathologic features, comorbidity scores, performance status and responses to the SF-36 Health Survey (SF-36) and Functional Assessment of Cancer Therapy-General (FACT-G) questionnaires between NSS and RN patients prior to surgery for newly diagnosed, localized RCC.

## Methods

### Nephrectomy registry

A valuable prerequisite for evaluating issues related to RCC survivorship is the availability of a large, comprehensively annotated database of patients undergoing curative surgical therapy for RCC. Related to this, we maintain a prospective Nephrectomy Registry database of all patients undergoing treatment for RCC at our institution.

Briefly, certified clinical coordinators consent and enroll patients to the Registry during the patient’s preoperative visit. After consent, the coordinators abstract over three hundred clinical variables from each patient’s medical record including demographic data, diagnosis date, inheritable syndromes, signs and symptoms, results of laboratory and imaging tests, performance status, surgical characteristics and surgical complications. In addition, the coordinators provide self-administered questionnaires to each enrolled patient at the time of enrollment to collect valuable lifestyle and risk factor data including smoking history, weight history, physician-diagnosed UTI and family history of cancer. Moreover, a urologic pathologist conducts a comprehensive, centralized review of all nephrectomy specimens to confirm several important prognostic features including histological subtype using the contemporary 1997 AJCC/UICC classification, tumor stage, nuclear grade, tumor size, and coagulative tumor necrosis. Most germane to this investigation, in 2007 the coordinators began requesting that all participants complete the modified FACT-G and the SF-36 QoL measures prior to surgery to establish a baseline measure of patient QoL metrics. This Registry effort provides the data on the target population of patients undergoing RN and NSS for our investigation. Of note, while over 95% of all patients who undergo surgical treatment for localized RCC at our institution consent to participate in our Registry, our response rate for the QoL questionnaire is approximately 49%. Interestingly, we note that responders tend to be slightly younger, have a higher body mass index, and have a better performance status as measure by the ECOG and Karnofsky measures when compared to non-responders (all p-values <0.05). In contrast, we noted no meaningful differences between responders and non-responders with regard to gender, education level, and race/ethnicity.

### Patient selection

Following approval by the Mayo Clinic Institutional Review Board (#610-05), we queried the Nephrectomy Registry and identified 144 patients treated with RN or NSS for unilateral, sporadic, RCC at our institution between May 2007 and November 2012 who completed both the SF-36 and FACT-G prior to their surgery.

### Clinical and pathologic features

We abstracted data from our Registry database on the following clinical variables: age at surgery, sex, comorbidity at presentation, ECOG and Karnofsky performance status, tumor thrombus level and Charlson score [11]. Patients with any component of the Charlson score, which includes history of myocardial infarction, congestive heart failure, peripheral vascular disease, cerebrovascular disease, dementia, chronic pulmonary disease, connective tissue disease, ulcer disease, mild liver disease, diabetes, hemiplegia, moderate or severe renal disease, diabetes with end organ damage, any previous tumor, leukemia, lymphoma, moderate or severe liver disease, any previous metastatic solid tumor, or AIDS were considered to have comorbid disease. In addition, we also abstracted data on the following pathologic features: tumor size, 2002 primary tumor classification, the 2002 TNM stage groupings, nuclear grade and presence of coagulative tumor necrosis. Of note, as part of our Registry effort, a single urologic pathologist (K.J.W.) completes a centralized review of all pathology specimens to confirm histologic diagnosis and provide robust pathologic metrics for our cases.

### Quality of life measures

As mentioned above, all 144 patients completed both the SF-36 Health Survey and the FACT-G questionnaire prior to surgical treatment for localized RCC. The SF-36 Health survey comprises 36 questions to assess 8 domains of functional health and well-being. The authors of the survey designed it to be used independent of age, disease, or treatment modality and as such it is widely used to assess QoL status in both general and disease-specific populations. All 36 questions on the SF-36 are scored on a scale from 0 to 100, with 100 as the highest level of functioning. Collective scores are calculated as a percentage of the total points possible. The scores from those questions that address each specific domain of functional health status are averaged together, for a final score with each of the 8 domains assessed. The FACT-G is a 33-item questionnaire that is a validated tool for assessing QoL associated with the management of chronic illness [12]. The questionnaire measures four specific QoL domains (physical, social, emotional, and functional well-being) which each have individual scores but are also summed to achieve a total score, with higher scores indicating better QoL. For this study, we used our experience from a previously published focus group of surgically-treated RCC patients to augment the FACT-G with eight additional questions designed to address kidney-specific QoL issues [4]. Each question presents a statement and the patient indicates whether this occurs “Not at All”, “A Little Bit”, “Somewhat”, “Quite a Bit” or “Very Much”.

### Statistical methods

For our analyses we summarized continuous data using the sample median (minimum, 25th percentile, 75th percentile, maximum) and categorical data as counts and percentages. For comparisons between our two surgical groups involving the median of continuous variables we employed Wilcoxon rank-sum tests and for comparisons involving percentages of categorical variables we utilized Fisher's Exact test. All statistical tests were two-sided, and the threshold of significance was set at p = 0.05.

## Results

A total of 144 patients were included in this study; 73 who underwent RN and 71 who underwent NSS. In Table 1, we provide a comparison of patient and RCC tumor characteristics between the two surgical groups. For demographic and anthropometric indices, patients undergoing RN were similar to NSS patients with regard to age, gender, marital status, education level; however, NSS patients were slightly more likely to be classified as obese given the higher percentage with a BMI greater than 30 kg/m^2^ (51% vs. 41% p = 0.07). Not surprisingly, we noted key differences in pathologic features with RN patients presenting with more aggressive tumors. Specifically, RN were more likely to be larger in size (5 cm vs. 3 cm; p <0.001), be grade 3 or 4 (39% vs. 11%; p =0.001) and classified as pT2 or later (40% vs. 5%; p <0.001). We observed no difference in ECOG or Karnofsky performance status between the RN and NSS groups (p = 0.76 and 0.62, respectively). While a global test suggested little evidence of an overall difference in Charlson score between the two groups (p = 0.18), it is worth noting that the percentage of patients with a Charlson score of 3 or more was notably higher in the RN compared to the NSS group (23% vs. 8%; p = 0.022). In Table 2 we provide a more detailed comparison of the patient symptoms at presentation for our two surgical groups. As expected, the RN group were more likely to present with several symptoms including discomfort on ipsilateral side (22% vs. 11%; p = 0.12), weight loss (10% vs. 1%; p = 0.063), gross hematuria (15% vs. 3%; p = 0.017) and impaired renal function (19% vs. 8%; p = 0.09).

**Table 1:** Patient characteristics by treatment type

***Variable***^***1***^	***NSS (N = 71)***	***RN (N = 73)***	***p-value***
*Age at diagnosis (years)*			0.28^*3*^
*< 65*	36 (51%)	34 (47%)	
*≥ 65*	35 (49%)	39 (53%)	
*Male*	45 (63%)	51 (70%)	0.48^*2*^
*Race - white*	65 (92%)	64 (88%)	0.59^*2*^
*Marital Status*			1.00^*2*^
*Married*	55 (77%)	57 (78%)	
*Widowed, Divorced, Single*	16 (23%)	16 (22%)	
*Education*			0.11^*2*^
*HS Graduate/GED*	8 (11%)	15 (21%)	
*1-3 Vocational/Some College*	30 (42%)	20 (27%)	
*College Graduate/Graduate School*	29 (41%)	29 (40%)	
*Other/Unknown*	4 (6%)	9 (12%)	
*BMI*			0.072^*3*^
*< 30*	35 (49%)	43 (59%)	
*≥ 30*	36 (51%)	30 (41%)	
*ECOG Performance Status*	n = 69	n = 72	0.76^*2*^
*0 – Fully active*	64 (93%)	65 (90%)	
*1, 2 – Some restriction*	5 (7%)	7 (10%)	
*Karnofsky Performance Status*	n = 62	n = 64	0.62^*3*^
*100*	55 (89%)	55 (86%)	
*70 - 90*	7 (11%)	9 (14%)	
*Histologic Subtype*			0.85^*2*^
*Clear cell*	46 (65%)	49 (67%)	
*Papillary/Chromophobe/RCC*	16 (23%)	17 (23%)	
*Oncocytoma/Angiomyolipoma/Other benign*	9 (13%)	7 (10%)	
*Nuclear grade*	n = 62	n = 66	0.001^*3*^
*1-2*	51 (82%)	40 (61%)	
*3-4*	11 (18%)	26 (39%)	
*Charlson score*			0.18^*3*^
*0*	37 (52%)	35 (48%)	
*1-2*	28 (39%)	21 (29%)	
*3-4*	6 (8%)	11 (15%)	
*≥ 5*	0 (0%)	6 (8%)	
*Tumor Stage*	n = 62	n = 65	<0.001^*2*^
*pT1a/pT1b*	59 (95%)	39 (60%)	
*pT2*	1 (2%)	10 (15%)	
*pT3a/pT3b*	2 (3%)	16 (25%)	

^*1*^The sample median (minimum, 25th percentile, 75th percentile, maximum) is given for numerical variables, n (%) for categorical variables.

^*2*^P-values result from Fisher’s exact test.

^*3*^P-values result from Wilcoxon rank sum test (although data is presented categorically).

**Table 2:** Patient symptoms at presentation in patients who completed a baseline QoL

***Variable***^***2***^	***Overall***^***1 ***^ ***(N = 144)***	***NSS***^***1 ***^ ***(N = 71)***	***RN***^***1 ***^ ***(N = 73)***
*Palpable flank*	2 (1%)	0 (0%)	2 (3%)
*Abdominal mass*	1 (1%)	0 (0%)	1 (1%)
*Discomfort ipsilateral side*	24 (17%)	8 (11%)	16 (22%)
*Discomfort contralateral side*	10 (7%)	2 (3%)	8 (11%)
*Rash*	1 (1%)	1 (1%)	0 (0%)
*Sweats*	5 (3%)	2 (3%)	3 (4%)
*Weight loss*	8 (6%)	1 (1%)	7 (10%)
*Fatigue*	7 (5%)	3 (4%)	4 (6%)
*GI early satiety or decreased appetite*	3 (2%)	0 (0%)	3 (4%)
*Gross hematuria*	13 (9%)	2 (3%)	11 (15%)
*Microhematuria*	17 (13%)	10 (15%)	7 (10%)
*Acute onset varicocele*	0 (0%)	0 (0%)	0 (0%)
*Impaired renal function*	20 (14%)	6 (8%)	14 (19%)
*Hypertension of recent onset*	0 (0%)	0 (0%)	0 (0%)
*Polycythemia of recent onset*	0 (0%)	0 (0%)	0 (0%)
*Anemia of recent onset*	0 (0%)	0 (0%)	0 (0%)
*Number of pre-existing conditions*			
*None*	69 (48%)	44 (62%)	25 (35%)
*1-2*	69 (48%)	27 (38%)	42 (58%)
*3+*	5 (3%)	0 (0%)	5 (7%)

^*1*^The n (%) is given for categorical variables.

^2^Information was unavailable for each symptom in a minimum of 1 patient to a maximum of 2 patients except for microhematuria which was unavailable in 9 patients.

In Table 3 we display a comparison of the scores on the SF-36 between our two surgical groups. Interestingly, despite the aforementioned differences in pathologic features and symptomatic presentation, NSS patients did not report significantly higher median scores on the SF-36 at baseline for the general health (72 vs. 67; p = 0.93) and vitality (53 vs. 50; p = 0.91) domains. We did observe evidence of considerably better median scores for NSS patients on the role physical (75 vs. 50; p = 0.50) domain; however this differences did not achieve conventional statistical significance. Interestingly, RN patients reported significantly higher median scores (i.e. better scores) on mental health (80 vs. 72; p = 0.023) domain. We noted similar trends for better scores for RN patients on the role emotional domain (100 vs. 67; p = 0.31) and bodily pain (71 vs. 62; p = 0.72) domains; however despite the large differences, the p-values could not rule out the role of chance as an explanation. In Table 4, we display a comparison of baseline scores on the FACT-G (which more closely reflects disease-specific QoL). In contrast to the evidence of differences on some domains of the SF-36, we observed strikingly similar responses on the FACT-G for the two surgical groups (i.e. all median scores within two points between the groups). Finally, in Table 5 we provide a comparison of the two groups across our additional questions that were added to the FACT-G based on results from the focus group [4]. Interestingly, we noted that “I experience significant pain in certain areas of my body” and “problems with pain limit my activities” was identified as happening “quite a bit or very much” more often in the NSS vs. the RN (29% vs. 22%; p = 0.42 and 20% vs. 10%, 0.18; respectively). As expected, our small sample size coupled with the multi-category nature of the responses to these statements (i.e. requiring more degrees of freedom in our statistical tests) limited our ability to rule out the role of chance.

**Table 3:** Patient Baseline SF-36 Index scores by treatment type (NSS vs. RN)

***Variable***	***NSS***^***1 ***^ ***(N = 71)***	***RN***^***1 ***^ ***(N = 73)***	***p-value***^***2***^
*Physical Functioning*	75 (5, 40, 95, 100)	75 (0, 50, 95, 100)	0.85
*Bodily Pain*	62 (0, 41, 88, 100)	71 (12, 41, 100, 100)	0.72
*General Health*	72 (10, 47, 82, 100)	67 (6.3, 52, 87, 100)	0.93
*Mental Health*	72 (20, 60, 84, 100)	80 (24, 64, 92, 100)	0.023
*Role Emotional*	67 (0, 0, 100, 100)	100 (0, 33, 100, 100)	0.31
*Role Physical*	75 (0, 0, 100, 100)	50 (0, 0, 100, 100)	0.50
*Social Functioning*	75 (0, 50, 100, 100)	75 (0, 50, 100, 100)	0.83
*Vitality*	53 (0, 30, 70, 100)	50 (0, 30, 75, 100)	0.91

^*1*^The sample median (minimum, 25th percentile, 75th percentile, maximum) is given.

^*2*^P-values result from Wilcoxon rank sum test comparing NSS to RN.

**Table 4:** Patient Baseline FACT-G Index scores by treatment type (NSS vs. RN)

***Variable***	***NSS***^***1 ***^ ***(N = 71)***	***RN***^***1 ***^ ***(N = 73)***	***p-value***^***2***^
*Physical Well Being*	25 (0, 20, 27, 28)	25 (5, 22, 27, 28)	0.90
*Emotional Well Being*	17 (5, 15, 19, 24)	18 (1, 14, 19, 24)	0.61
*Social/Family Well Being*	25 (0, 21, 27, 28)	25 (9, 21, 28, 28)	0.71
*Functional Well Being*	21 (2.3, 16, 24, 28)	19 (5, 14, 24, 28)	0.33
*Total FACT-G*	84 (39, 74, 93, 107)	81 (38, 70, 96, 107)	0.72

^*1*^The sample median (minimum, 25th percentile, 75th percentile, maximum) is given.

^*2*^P-values result from Wilcoxon rank sum test comparing NSS to RN.

**Table 5:** Baseline Reponses to RCC-specific QoL Questions Added to the FACT-G by treatment type (NSS vs. RN)

***Variable***^***3***^	***NSS***^***1 ***^ ***(N = 71)***	***RN***^***1 ***^ ***(N = 73)***	***p-value***^***2***^
*I urinate more frequently than usual*			0.90
*Not at all & A little bit*	41 (59%)	40 (56%)	
*Somewhat*	16 (23%)	19 (26%)	
*Quite a bit & Very much*	13 (19%)	13 (18%)	
*I have sudden strong urges to urinate*			0.28
*Not at all & A little bit*	41 (59%)	47 (65%)	
*Somewhat*	16 (23%)	9 (13%)	
*Quite a bit & Very much*	13 (19%)	16 (22%)	
*Problems with urinating limit my activities*			1.00
*Not at all & A little bit*	62 (89%)	63 (89%)	
*Somewhat*	6 (9%)	7 (10%)	
*Quite a bit & Very much*	2 (3%)	1 (1%)	
*I have been vomiting*			0.24
*Not at all & A little bit*	66 (94%)	70 (97%)	
*Somewhat*	1 (1%)	2 (3%)	
*Quite a bit & Very much*	3 (4%)	0 (0%)	
*Injuries (cuts, scrapes, bruises) heal slowly*			0.83
*Not at all & A little bit*	58 (83%)	62 (86%)	
*Somewhat*	7 (10%)	6 (8%)	
*Quite a bit & Very much*	5 (7%)	4 (6%)	
*I experience significant pain in certain areas of my body*			0.42
*Not at all & A little bit*	41 (59%)	50 (69%)	
*Somewhat*	9 (13%)	6 (8%)	
*Quite a bit & Very much*	20 (29%)	16 (22%)	
*Problems with pain limit my activities*			0.18
*Not at all & A little bit*	49 (70%)	54 (75%)	
*Somewhat*	7 (10%)	11 (15%)	
*Quite a bit & Very much*	14 (20%)	7 (10%)	
*I am satisfied with my present comfort level*			0.54
*Not at all & A little bit*	21 (30%)	20 (28%)	
*Somewhat*	21 (30%)	17 (24%)	
*Quite a bit & Very much*	28 (40%)	35 (49%)	
*I am forgetful*			0.27
*Not at all & A little bit*	49 (70%)	41 (57%)	
*Somewhat*	14 (20%)	19 (26%)	
*Quite a bit & Very much*	7 (10%)	12 (17%)	

^*1*^N (%) is given.

^*2*^P-values result from Fisher’s Exact test comparing association of Not at all & A little bit, Somewhat, Quite a bit & Very much (3 groups) to Partial and Radical surgical groups.

^*3*^Information was not available for one patient in the NSS group and 1 to a maximum of 2 patients in the RN group.

Finally, given the differences we noted in pathologic features between the RN and NSS groups (particularly pT stage), in an exploratory fashion we repeated our comparisons limiting to only those patients with pT1 disease (NSS = 59 pts, RN = 39 pts) and present these data in Tables 6, 7 and 8. While many of the results for the SF-36 were unchanged in this subgroup analysis, the evidence of higher scores at baseline for NSS on the Role Physical (100 vs. 25; p = 0.08) and Vitality (60 vs. 50; p = 0.49) domains strengthened slightly (Table 6). The results for the standard FACT-G (i.e. very similar median scores for NSS and RN across all domains) remained unchanged in subset of patients with pT1 disease (Table 7). The slight differences between NSS and RN with regard to “I experience significant pain” and “problems with pain limits my activities” that we noted for the entire cohort were mitigated in the subset of pT1 patients (26% vs. 26%; p0.74 and 17% vs. 13% p = 0.65; respectively; Table 8).

**Table 6:** Patient Baseline SF-36 Index scores by treatment type (NSS vs. RN) in patients with pT1 RCC

***Variable***	***NSS***^***1 ***^ ***(N = 59)***	***RN***^***1 ***^ ***(N = 39)***	***p-value***^***2***^
*Physical Functioning*	75 (5, 37.5, 95, 100)	68 (0, 50, 85, 100)	0.51
*Bodily Pain*	62 (0, 41, 86, 100)	51 (20, 31, 74, 100)	0.35
*General Health*	72 (10, 50, 82, 100)	77 (6.3, 37, 90, 100)	0.91
*Mental Health*	72 (35, 60, 84, 100)	80 (24, 64, 96, 100)	0.059
*Role Emotional*	67 (0, 0, 100, 100)	100 (0, 0, 100, 100)	0.64
*Role Physical*	100 (0, 0, 100, 100)	25 (0, 0, 100, 100)	0.081
*Social Functioning*	75 (0, 56.3, 100, 100)	75 (12.5, 37.5, 100, 100)	0.68
*Vitality*	60 (0, 35, 70, 100)	50 (5, 30, 70, 100)	0.49

^*1*^The sample median (minimum, 25th percentile, 75th percentile, maximum) is given.

^*2*^P-values result from Wilcoxon rank sum test comparing NSS to RN.

**Table 7:** Patient Baseline FACT-G Index scores by treatment type (NSS vs. RN) in patients with pT1 RCC

***Variable***	***NSS***^***1 ***^ ***(N = 59)***	***RN***^***1 ***^ ***(N = 39)***	***p-value***^***2***^
*Physical Well Being*	26 (0, 20, 27, 28)	24 (4.7, 19, 27, 28)	0.39
*Emotional Well Being*	17 (8, 15, 19, 24)	18 (10, 14, 20, 24)	0.35
*Social/Family Well Being*	24 (0, 20, 27, 28)	24 (10, 19, 26, 28)	0.73
*Functional Well Being*	21 (2.3, 16, 24, 28)	18 (5, 14, 24, 28)	0.29
*Total FACT-G*	83 (39, 74, 93, 104)	80 (37.7, 69, 94, 105)	0.48

^*1*^The sample median (minimum, 25th percentile, 75th percentile, maximum) is given.

^*2*^P-values result from Wilcoxon rank sum test comparing Partial to Radical.

**Table 8:** Baseline Reponses to RCC-specific Questions Added to the FACT-G by treatment type (NSS vs. RN) in patients with pT1 RCC

***Variable***	***NSS***^***1 ***^ ***(N = 59)***	***RN***^***1 ***^ ***(N = 39)***	***p-value***^***2***^
*I urinate more frequently than usual*			0.44
*Not at all & A little bit*	35 (60%)	20 (53%)	
*Somewhat*	13 (22%)	13 (34%)	
*Quite a bit & Very much*	10 (17%)	5 (13%)	
*I have sudden strong urges to urinate*			0.87
*Not at all & A little bit*	35 (60%)	24 (63%)	
*Somewhat*	12 (21%)	6 (16%)	
*Quite a bit & Very much*	11 (19%)	8 (21%)	
*Problems with urinating limit my activities*			0.68
*Not at all & A little bit*	52 (90%)	33 (89%)	
*Somewhat*	6 (10%)	3 (8%)	
*Quite a bit & Very much*	0 (0%)	1 (3%)	
*I have been vomiting*			0.49
*Not at all & A little bit*	55 (95%)	36 (95%)	
*Somewhat*	1 (2%)	2 (5%)	
*Quite a bit & Very much*	2 (3%)	0 (0%)	
*Injuries (cuts, scrapes, bruises) heal slowly*			0.77
*Not at all & A little bit*	49 (84%)	30 (79%)	
*Somewhat*	6 (10%)	5 (13%)	
*Quite a bit & Very much*	3 (5%)	3 (8%)	
*I experience significant pain in certain areas of my body*			0.74
*Not at all & A little bit*	35 (60%)	25 (66%)	
*Somewhat*	8 (14%)	3 (8%)	
*Quite a bit & Very much*	15 (26%)	10 (26%)	
*Problems with pain limit my activities*			0.65
*Not at all & A little bit*	41 (71%)	26 (68%)	
*Somewhat*	7 (12%)	7 (18%)	
*Quite a bit & Very much*	10 (17%)	5 (13%)	
*I am satisfied with my present comfort level*			1.00
*Not at all & A little bit*	18 (31%)	12 (32%)	
*Somewhat*	17 (29%)	11 (29%)	
*Quite a bit & Very much*	23 (40%)	15 (39%)	
*I am forgetful*			0.093
*Not at all & A little bit*	43 (74%)	20 (53%)	
*Somewhat*	10 (17%)	11 (29%)	
*Quite a bit & Very much*	5 (9%)	7 (18%)	

^*1*^N (%) is given.

^*1*^Additional Fact-G questions were not responded to in n = 1 NSS and n = 1 RN patient.

^*2*^P-values result from Fisher’s Exact test comparing association of ‘Not at All & A Little Bit’, ‘Somewhat’, ‘Quite a Bit & Very Much’ (3 groups) to NSS and RN groups.

## Discussion

The number of patients living for long periods of time following surgical excision of a clinically localized RCC has increased over the past three decades. As such, issues of survivorship have moved closer to the forefront of research efforts. Related to this, studies depicting similar oncological and QoL outcomes for NSS and RN have been used to underscore the importance of the adoption of NSS in eligible patients with RCC [2]. The demonstrated value of NSS notwithstanding, what has been lacking in the discussion to this point has been a direct comparison of baseline characteristics (including QoL measures) between patients electing to undergo NSS versus RN for localized RCC. That is, to date there are no published data to suggest that RN and NSS patients have similar QoL status at baseline (an assumption that is inherent in the existing studies that have compared QoL outcomes between the two groups). If baseline differences do exist, this could have implications on the interpretation of follow-up QoL data. By example, if RN patients have higher baseline QoL on average compared to NSS patients, then a study reporting similar QoL metrics at follow-up for these two groups would mask the fact that the surgery had a greater negative impact on the RN versus the NSS group.

To address the gap in our knowledge regarding baseline comparisons of between NSS and RN patients, we used data from our prospective Registry to report for the first time on simultaneous comparisons of baseline demographics, pathologic features, performance scores, comorbidities, symptoms at presentation and responses to QoL surveys between NSS and RN patients. Specifically, we observed RN patients to have more aggressive tumor pathology and a slightly lower BMI compared to NSS patients. While we noted no differences in overall performance scores between the two groups, we did observe that RN patients were more likely to be classified in the highest Charlson score categories (i.e. > = 3) and report a variety of symptoms at presentation than the NSS patients. From a QoL perspective, we report evidence of differences between RN and NSS patients scores on the standard SF-36 and a set of amended questions to the FACT-G that were developed from our previous focus group of RCC patients. [4] Interestingly, we report no evidence of differences between the two groups on the FACT-G. When we analyzed only the subset of RCC patients in each surgical group with pT1 disease (i.e. the group most likely to receive NSS), we observed emerging evidence of better baseline scores for the NSS compared to RN patients on the Role Physical and Vitality domains of the SF-36. In addition, we also observed significantly lower baseline scores on the Mental Health domain in the NSS group compared to the RN group. In contrast, scores on the standard FACT-G remained similar and the differences we noted on the set of amended RCC-specific questions were attenuated.

This contrast between the results we obtained on the SF-36 and the FACT-G warrants further discussion, with a likely explanation centering on the nature of the surveys. That is, the SF-36 is designed to measure general well-being, while the FACT-G is more disease-specific (to RCC, in our case). For example, the SF-36 Mental Health domain is intended to evaluate the general emotional state of the individual (high score implies feeling peaceful, happy, calm) unrelated to any disease state. By comparison, the Emotional Well-being domain of the FACT-G is intended to assess the emotional state of the individual in terms of their cancer (e.g., “I am satisfied with how I am coping with my illness”). With this in mind, a logical interpretation of our results is that the QoL metrics that are important for patients with RCC, and those that could be modulated by the type of surgery are not disease specific. Further supporting this notion is our own previous observation that RCC patients do not express a high level of concern regarding their cancer following surgical excision of the tumor [4]. Indeed, attitudes expressed in the focus groups for our previous study indicated a low level of concern regarding the risk of cancer recurrence following surgery. Therefore, in contrast to patients with breast and prostate cancer who often express a high level of anxiety about their risk of recurrence following surgical treatment, patients with RCC are comparatively less focused on cancer-related issues due in part to a feeling of being “cured”. This is consistent with our observation in the current study that the disparities in QoL measures between NSS and RN are limited to only non-disease specific metrics measured by the more general SF-36.

In our review of the literature, we identified seven teams of investigators who prior to 2012 reported on QoL metrics in RCC patients post surgical intervention. Of these, three provided analysis directly comparing QoL outcomes between NSS and RN patients. Interestingly, while Poulakis et al. [5] concluded that there was no difference in follow-up QoL measures between the NSS and RN groups, both Clark and Ficarra present data suggesting that QoL outcomes may be better among the NSS group [6,8]. The strengths of these two studies aside, it is worth noting that neither team conducted prospective enrollment of patients, the time to follow-up QoL measures varied considerably across participants in each study and most importantly, neither provided direct comparisons of preexisting comorbidities, symptoms at presentation or baseline measures of QoL between the RN and NSS groups. As such, given that the absence of a baseline for each group, it is difficult to draw meaningful conclusions regarding the true effect of NSS and RN on RCC patient QoL.

More recently, Parker et al. [10]. advanced the field with their publication of data from the most comprehensive, prospective assessment to date of QoL outcomes in patients undergoing surgery for localized RCC. Briefly, the authors report comparisons of QoL outcomes data on 172 patients undergoing surgery for localized RCC by one of four surgical modalities (laparoscopic partial = 20, laparoscopic radical =55, open partial = 72, and open radical =25). Of interest to our findings, the authors report that those patients undergoing RN reported lower global scores on the CARES-SF survey at all follow-up time points (indicating better cancer-specific QoL) than patients who underwent NSS. While these results have advanced our overall understanding of the impact of RCC surgery on patient QoL, there are key aspects of this study that should be considered before drawing final conclusions. First and foremost, while the authors did collect baseline QoL data they do not present these data for review and only state that they observed “no significant differences in baseline scores”. Given that the focus of the paper was on four surgical modalities, without a display of the baseline data it is difficult to know if specific differences between the RN and NSS subgroups may have existed but were not statistically significant due to a global statistical test across four patient subgroups (instead of just NSS vs. RN). Moreover, in Figure B of their manuscript they display a graph showing notable differences in QoL scores between RN and NSS groups at the first 3 week follow-up time point, thus underscoring the question of how comparable these two groups were with respect to QoL metrics at baseline. In addition to the absence of a presentation of the baseline QoL scores, Parker et al. do not provide any data on the relevant issues of performance status, comorbidity or symptoms at presentation for the surgical groups at baseline. Related to this, our findings regarding Charlson Score suggest that the RN patients are more likely to have higher Charlson scores (i.e. 3 or greater) at baseline than the NSS group. Similarly, we assessed patient symptoms at presentation and report that RN patients had higher incidence of symptoms at presentation than NSS patients. As such, our data support that both of these factors (increased comorbid disease and greater symptoms at baseline) can and should be factored in to analysis of QoL outcomes between these two surgical groups.

There are specific limitations of the present study to be mindful of when interpreting our results, chief among these being our limited sample size. More specifically, we must be mindful that due to the smaller sample size in this investigation, our power to detect potentially meaningful clinical differences is limited. As such, we would advocate against the dismissal of a difference in scores that is notable but does not have an adequate p-value to achieve conventional statistical significance, since this could simply be due to the limited power of this pilot study. The limited power in certain cases notwithstanding, ours is the first study to directly compare baseline metrics between NSS and RN patients and as such, our results are informative to the larger discussion of the need to include baseline data when comparing QoL outcomes between these two patient groups. Related to this, our sample is drawn from a patient population at a tertiary referral center, and therefore may lack generalizability to the general population of RCC patients. As such, validation of our results in a larger, more diverse patient group is needed. Specifically, a prospective evaluation of health-related QoL outcomes that includes baseline measures with larger numbers in both surgical groups would serve as a validation study in this population. Related to this, we advocate for efforts to centralize the collection of QoL data in a more systematic way across institutions like ours that maintain prospective RCC registries. Such efforts would greatly enhance large-scale validation opportunities. Finally, we did not use a validated survey for measuring RCC-specific QoL because there are currently no validated tools for measuring RCC-specific QoL. We attempt to address this disparity by utilizing a modified, renal specific version of the FACT-G that included questions derived from our previously published focus group of RCC patients [4]. The benefits of this approach aside, future investigations of RCC-related QoL can and should address this need for a validated measure as this will be of great importance in an era where issues of survivorship have moved closer to the forefront of research efforts.

## Conclusions

We report evidence of differences in key clinical and pathological metrics at baseline between patients undergoing NSS and RN. We also report for the first time that RN patients indicate better mental health status on the SF-36 than NSS patients at baseline. The remainder of the differences we noted on the SF-36 domains achieved only borderline statistical significance and we report no evidence of differences on the standard FACT-G. That being said, our data support the need for consideration of baseline status when conducting comparisons of follow-up QoL measures between these two surgical groups. More importantly, our report underscores the need for the development of better, more-kidney-specific QoL assessment tools in order to accurately reflect any post-operative change in QoL as it relates to surgical treatment.

## Abbreviations

BMI: Body mass index; NSS: Nephron-sparing surgery; QoL: Quality of Life; RCC: Renal cell carcinoma; RN: Radical nephrectomy.

## Competing interests

AP is an associate editor for *BMC Urology*. The authors have nothing else to disclose.

## Authors’ contributions

MA – study conception and design, literature search, clinical studies, data acquisition, manuscript preparation, editing, review, final approval of the manuscript. DT – study conception, literature search, clinical studies, manuscript preparation, editing, review, final approval of the manuscript. ND – study design, data analysis, statistical analysis, manuscript editing, review, final approval of the manuscript. KW – study design, data acquisition, manuscript editing, review, final approval of the manuscript. SA – study design, literature search, manuscript editing, review, final approval of the manuscript. AP – study conception and design, literature search, clinical studies, data acquisition, data analysis, statistical analysis, manuscript preparation, editing, review, final approval of the manuscript, guarantor of the manuscript. All authors read and approved the final manuscript.

## Pre-publication history

The pre-publication history for this paper can be accessed here:

http://www.biomedcentral.com/1471-2490/13/52/prepub
